# Insights Into the Metabolic Aspects of Aortic Stenosis With the Use of Magnetic Resonance Imaging

**DOI:** 10.1016/j.jcmg.2022.04.025

**Published:** 2022-12

**Authors:** Shveta Monga, Ladislav Valkovič, Damian Tyler, Craig A. Lygate, Oliver Rider, Saul G. Myerson, Stefan Neubauer, Masliza Mahmod

**Affiliations:** a Division of Cardiovascular Medicine, Radcliffe Department of Medicine, University of Oxford, Oxford, United Kingdom; b Department of Imaging Methods, Institute of Measurement Science, Slovak Academy of Sciences, Bratislava, Slovakia; c Department of Physiology, Anatomy and Genetics, University of Oxford, Oxford, United Kingdom; d Wellcome Centre for Human Genetics, Oxford, United Kingdom

**Keywords:** aortic stenosis, cardiac magnetic resonance, magnetic resonance spectroscopy, myocardial metabolism, ADP, adenosine diphosphate, ATP, adenosine triphosphate, CK, creatine kinase, FAO, fatty acid oxidation, HF, heart failure, LVH, left ventricular hypertrophy, MRS, magnetic resonance spectroscopy, MTG, myocardial triglyceride, PCr, phosphocreatine, PDH, pyruvate dehydrogenase, PPAR, peroxisome proliferator–activated receptor, TCA, tricarboxylic acid

## Abstract

Pressure overload in aortic stenosis (AS) encompasses both structural and metabolic remodeling and increases the risk of decompensation into heart failure. A major component of metabolic derangement in AS is abnormal cardiac substrate use, with down-regulation of fatty acid oxidation, increased reliance on glucose metabolism, and subsequent myocardial lipid accumulation. These changes are associated with energetic and functional cardiac impairment in AS and can be assessed with the use of cardiac magnetic resonance spectroscopy (MRS). Proton MRS allows the assessment of myocardial triglyceride content and creatine concentration. Phosphorous MRS allows noninvasive in vivo quantification of the phosphocreatine-to-adenosine triphosphate ratio, a measure of cardiac energy status that is reduced in patients with severe AS. This review summarizes the changes to cardiac substrate and high-energy phosphorous metabolism and how they affect cardiac function in AS. The authors focus on the role of MRS to assess these metabolic changes, and potentially guide future (cellular) metabolic therapy in AS.

## Highlights

• Understanding the cellular pathophysiologic processes in ASmay help to identify patients likely to decompensate early, and to explore potential therapeutic targets that could delay disease progression.
• Altered cardiac substrate utilization and consequent myocardial steatosis and reduced energy efficiency has been implicated in the transition from compensated hypertrophy to heart failure in AS.
• Magnetic resonance spectroscopy allows detailed assessment of changes to cardiac substrate and high-energy phosphorous metabolism, improving our understanding of the links between abnormal metabolism and impairment of cardiac function in AS.

Aortic stenosis (AS) is a common cardiovascular disorder, with an estimated prevalence of approximately 2% among individuals aged 65 to 70 years, increasing to 3% to 9% after the age of 80 years.^1^ This presents an increasing societal and economic burden. Current guidelines recommend aortic valve replacement as the definitive treatment for severe AS, but only after the onset of clinical symptoms or when there is impaired left ventricular (LV) systolic function. Therapeutic alternatives to valve replacement are extremely limited, particularly those to aid the myocardium cope better with AS. There is also no treatment for asymptomatic moderate or severe AS with preserved systolic function, and patients currently wait until valve replacement is warranted, that is, as an end-stage mechanical option.

**Figure 1:**
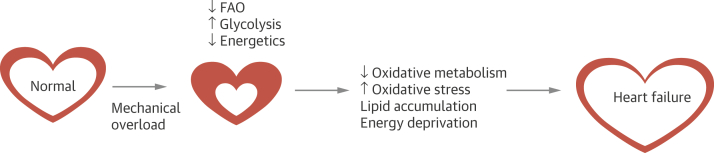
Schematic Representation of Metabolic Remodeling and the Development of Heart Failure in Aortic Stenosis Pathologic hypertrophy in response to mechanical overload, as in aortic stenosis, is accompanied by metabolic remodeling characterized by decreases in fatty acid oxidation (FAO) and increases in glycolysis. This fetal-like metabolic profile decreases the capacity for adenosine triphosphate synthesis, which is consistent with the energy starvation model. Persistent metabolic derangements elicit decreased oxidative metabolism, increased oxidative stress, lipid accumulation, and energy deprivation, all contributing to the progression of heart failure.

Understanding the metabolic and physiologic pathways in AS may identify suitable targets for future treatments that could provide alternatives to end-stage valve replacement. Ongoing pressure overload in AS increases myocardial wall stress and leads to an increase in wall thickness and mass, which results in left ventricular hypertrophy (LVH).^2^ Pathologic LVH in AS appears to be a typical cardiac phenotypic response to stress, encompassing structural and metabolic remodeling eventually leading to a cardiomyopathy-like process with impaired myocardial metabolism and energetics (Figure 1). Identifying early markers of cardiac decompensation would help to identify those most at risk of transition to heart failure (HF).

In this review, we discuss the metabolic alterations that occur in AS and the potential links between abnormal metabolism and progression from compensated (“appropriate physiologic”) hypertrophy to HF. We focus on the role of magnetic resonance (MR) metabolic imaging in detecting these changes (Central Illustration) and introduce the subject of metabolic modulation as a potential therapeutic option in AS.

**Central Illustration:**
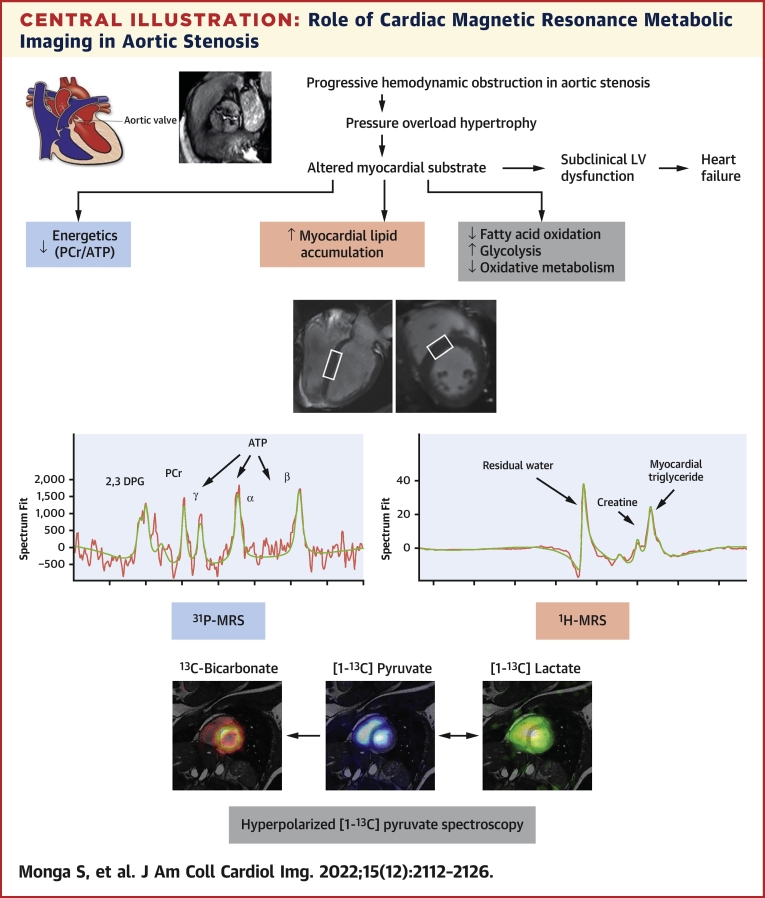
Role of Cardiac Magnetic Resonance Metabolic Imaging in Aortic Stenosis An overview of the various cardiac magnetic resonance metabolic imaging techniques and their ability to study specific aspects of cardiac metabolism in aortic stenosis. ^1^H = proton; ^13^C = carbon; ^31^P = phosphorous; ATP = adenosine triphosphate; DPG = diphosphoglycerol; LV = left ventricular; MRS = magnetic resonance spectroscopy; PCr = phosphocreatine.

## Normal Cardiac Metabolism

**Figure 2:**
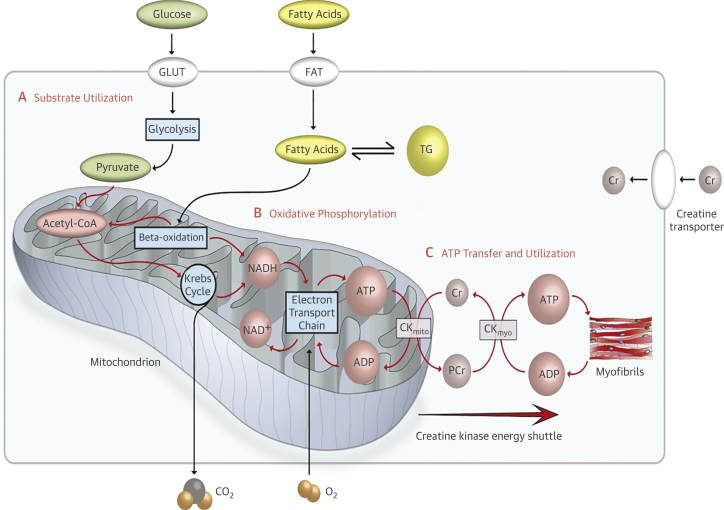
Normal Cardiac Metabolism, Illustrating 3 Main Steps in Energy Production **(A)** Substrate utilization. **(B)** Oxidative phosphorylation. **(C)** Adenosine triphosphate (ATP) transfer and utilization. Myocardial triglyceride content (TG) and total creatine (Cr) can be measured with the use of ^1^H magnetic resonance spectroscopy (MRS). Phosphocreatine (PCr) and ATP can be assessed with the use of ^31^P-MRS. Acetyl-CoA = acetyl coenzyme A; ADP = adenosine diphosphate; CK_mito_ = mitochondrial creatine kinase; CK_myo_ = myofibrill creatine kinase; FAT = fatty acid transporter; GLUT = glucose transporter; NAD^+^ = nicotinamide adenine dinucleotide; NADH = reduced nicotinamide adenine dinucleotide. From Masliza Mahmod, DPhil thesis 2013.

As a continually working aerobic biological pump, the adult human heart has the highest energy demand for adenosine triphosphate (ATP) per gram weight of any organ: around 6 kg daily, which is 15-20 times its own weight.^3^,^4^ Normal cardiomyocyte metabolism (Figure 2) comprises 3 key stages. The first stage is substrate utilization, that is, cellular uptake of substrates followed by their breakdown via metabolic pathways, such as beta-oxidation and glycolysis, to generate acetyl coenzyme A (acetyl-CoA) which then enters the tricarboxylic acid or Krebs cycle. In the adult heart, fatty acids (FAs) are the main energy source, accounting for 60% to 90%, and the remaining 10% to 40% comes from glucose, amino acids, pyruvate, lactic acid, ketone bodies, and other sources.^5^,^6^ The second stage is oxidative phosphorylation, that is, the process in which the high-energy phosphate compound, ATP, is formed through phosphorylation of adenosine diphosphate (ADP) in the inner mitochondrial membrane as a result of the transfer of electrons from the reduced NADH/FADH_2_, produced in beta-oxidation, glycolysis, and the Krebs cycle, to O_2_ by series of electron carriers. The third component is ATP transfer and utilization, that is, the transport of energy to, and its consumption by, the myofibrils. This is facilitated through an energy-transfer mechanism termed the creatine kinase (CK) energy shuttle.^7^ The CK system plays an important role in myocardial energy metabolism by maintaining ADP levels high in the mitochondria (CK mitochondrial isoform), where ATP is generated, and low at sites of ATP utilization (CK muscle isoform), thereby enhancing the efficiency of the energy utilization processes (Figure 2).^8^ The phosphocreatine (PCr)/ATP ratio is one indicator of this energetic state of the myocardium and is reduced in hypertrophied hearts^9^ and in HF.^10^ However, PCr/ATP ratio does not directly reflect the rate of ATP production through the CK reaction. ATP levels fall only when PCr levels are substantially depleted, because the CK system strongly favors ATP synthesis above PCr synthesis, which may be more important in the progression to HF in patients with LVH.^11^

**Figure 3:**
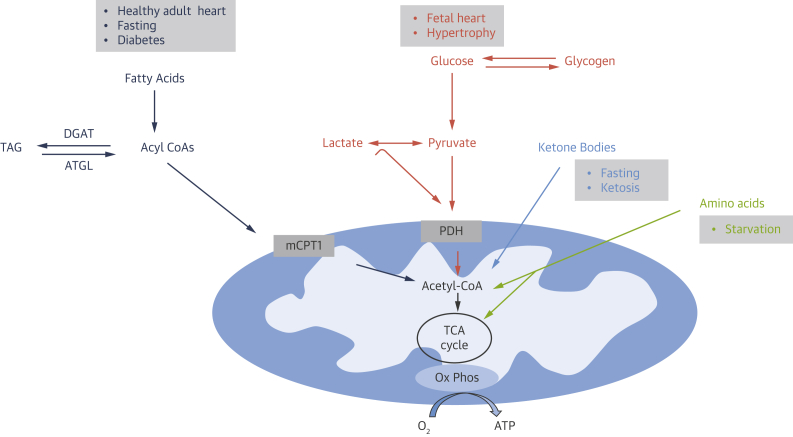
The Energy-Yielding Substrates (Fatty Acids, Glucose, Ketones, and Amino Acids), via Specific Catabolic Pathways, Converge on Acetyl-CoA Production With Subsequent Entry Into the TCA Cycle The final step of energy transfer is accomplished through oxidative phosphorylation (Ox Phos), supplying >95% of ATP consumed by the heart. The **boxes** above each metabolic pathway indicate the pathologic and physiologic condition in which the specific substrate becomes a predominant contributor to metabolism. ATGL = adipose triglyceride lipase; DGAT = diacylglycerol acyltransferase; mCPT1 = muscle form of carnitine-palmitoyl transferase 1; PDH = pyruvate dehydrogenase; TAG = triacylglycerol; TCA = tricarboxylic acid; other abbreviations as in Figure 2.

The metabolic flexibility of the heart allows it to consume nearly all types of energy substrates to form ATP,^6^ determined by external factors such as the availability of substrates in the blood^12^ or pathology^13^ (Figure 3). Apart from substrate availability, other complex regulatory mechanisms, including transcriptional regulation and posttranslational modification of key proteins, contribute to metabolic flexibility at multiple levels in each metabolic pathway. The balance between cellular energy metabolism and contractile performance is disrupted in cardiac disease.^7^

## Metabolic Alterations in AS and Their Assessment With the Use of MRI

### Substrate selection

Hypertrophied hearts in AS undergo a shift in substrate utilization similar to that seen in fetal hearts, with down-regulation of fatty acid oxidation (FAO) and increased reliance on glucose.^14^ This increased glucose use is predominantly characterized by increased glucose uptake and glycolysis with either no change or a decrease in glucose oxidation.^15^^,^^16^ The reliance on glycolysis combined with possible up-regulation of intermediary metabolism to maintain tricarboxylic acid (TCA) flux^17^^,^^18^ slightly improves myocardial oxygen efficiency, but whether the response varies with the severity of AS and becomes maladaptive with HF progression is unclear. Recent studies have also shown increased cardiac uptake of ketone bodies in AS-induced LVH,^19^ which may still only be a minor fuel source having increased uptake from a very low basal level. Whether this increased utilization of ketone bodies is adaptive or maladaptive and how that interacts with FA and glucose metabolism are currently unclear, but data in the literature support a potential beneficial effect of ketone body metabolism in HF with reduced ejection fraction.^20^

The knowledge of such metabolic interactions in AS and their role in transition to HF could potentially help to risk-stratify individuals and thus be of significant clinical value.

### Lipid metabolism in hypertrophied heart

FAs utilized for cardiac FA beta-oxidation primarily originate from either circulating nonesterified FAs bound to albumin (free FA)^3^ or from esterified FAs contained within lipoprotein-derived triacylglycerols. The majority of free FA molecules are oxidized in the mitochondria to deliver energy for cardiac electromechanical activity and other ATP-requiring processes. Remaining unused, free FAs are incorporated into esterified FA pools such as triacylglycerols, phosphoglycerides, and cholesteryl esters.

In pressure-overload hypertrophy, dysregulated FAO with a shift in substrate utilization causes an imbalance between FA uptake and oxidation giving rise to myocardial lipid accumulation/steatosis. Because cardiomyocytes are not specialized to store lipids, the excess accumulated intramyocardial free FAs enter nonoxidative pathways and are transformed into toxic intermediates such as diacylglycerols and ceramides.^21^ In nonhuman animal models, these intermediates compromise ATP production and overall cell viability and have been associated with increased oxidative stress, apoptosis, and cardiac dysfunction.^4^,^20^ As the heart shifts from compensated hypertrophy to HF, these toxic metabolites can alter gene expression by means of nuclear-receptor interaction and can stimulate apoptotic signal transduction pathways. This can lead to an increase in mitochondrial-uncoupling cardiac proteins, which sequentially are associated with decreased mitochondrial respiratory coupling and low cardiac efficiency.^22^^,^^23^

**Figure 4:**
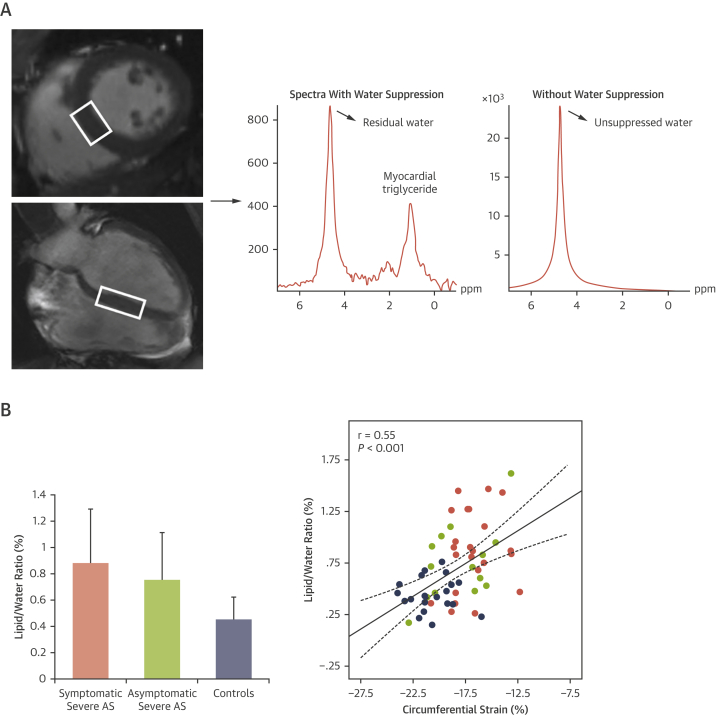
Myocardial Lipid Content ^1^H Spectrum of the Heart **(A)** Images on the **left** show the typical position of voxel in the interventricular septum. Spectra with and without water suppression are acquired as shown on the **right.** Myocardial triglyceride (MTG) is then calculated as the sum of detected lipid signal amplitudes (=CH-CH_2_-CH= @ 2.2 ppm, -CH_2_- @ 1.3 ppm, -CH_3_ @ 0.9 ppm)/water signal amplitude × 100. **(B)** Graph on the **left** compares the myocardial lipid content in symptomatic severe aortic stenosis (AS), asymptomatic severe AS, and healthy control subjects. Graph on the **right** depicts relationship between myocardial steatosis (as measured by ^1^H-MRS) and left ventricular strain in asymptomatic and symptomatic AS patients. Figure 4B reproduced with permission from Mahmod et al.^28^

Multiple preclinical studies suggest a causal link between steatosis and LV dysfunction demonstrating that mismatch between myocardial FA uptake and utilization can lead to cardiac lipotoxicity and lipid-induced programmed cell death.^24^^,^^25^ This occurs after lipid accumulation and before the development of significant LV dysfunction.^24^ A causal relationship is further supported by the fact that the antisteatotic agent, troglitazone, has been shown to reduce cardiac triglyceride content, and prevent both cardiac apoptosis and loss of myocardial function in obese rat models.^26^ Marfella et al^27^ have shown that altered myocardial substrate utilization and consequent steatosis causes LV dysfunction in patients with AS and metabolic syndrome. Our group has shown that steatosis is present in severe AS and independently correlates with LV dysfunction as measured by myocardial strain parameters (Figure 4B). Importantly, following valve replacement, there is both regression of steatosis and improvement in myocardial strain.^28^

Thus, the evidence so far supports the hypothesis of substrate switch, lipid overload, and subsequent mitochondrial dysfunction and contractile impairment in AS. However, most of the data to date are from nonhuman animal models of aortic banding rather than the real-world insulin-resistant human population with chronic AS.^29^ This further highlights the need for imaging techniques that can help assess these important metabolic alterations in the real-world AS population and guide future strategies to risk stratify and better manage this condition.

Magnetic resonance spectroscopy (MRS) is the only noninvasive, non–radiation-exposure technique for the investigation of cardiac metabolism in vivo.^30^ The basic principle enabling MRS is that the distribution of electrons within an atom cause nuclei in different chemical environments to experience slightly different magnetic fields. This results in different resonant frequencies, which in turn cause signals from these nuclei to appear as separate peaks in the MR spectrum.^31^ MRS uses MR signals from nuclei, such as ^31^phosphorus, ^1^hydrogen, ^13^carbon, and ^23^sodium, to provide comprehensive metabolic and biochemical information about cardiac muscle. This method is highly versatile and can provide metabolic insights into the role of cardiac metabolism in a wide number of conditions, including hypertensive, valvular, and ischemic heart disease, heart failure, and other cardiomyopathies. This method can also be used to monitor patient responses to therapeutic interventions: pharmacologic,^32^ surgical, or interventional.^33^ When combined with cardiovascular MRI, MRS enables detailed pathophysiologic insights into the interrelations among cardiac structure, function, and metabolism. However, MRS is currently used primarily as a research tool because of low spatial resolution and reproducibility.

Cardiac MRS uses mostly the same hardware as conventional cardiac magnetic resonance (CMR) for patients, typically a 1.5-T or 3.0-T magnet (ultrafield [7.0-T to 18.0-T] for experimental studies), with additional hardware including nucleus-specific coils (eg, ^31^P-coil) and a broadband radiofrequency transmitter to excite nonproton nuclei. Specific MRS acquisition sequences, MRS postprocessing, and data analysis packages are also required.

MRS holds great promise as a clinical tool in the near future, but will require development in technique, equipment, and expertise. Recent progress in the research community is helping to address these issues, but a major disparity remains between what is available for research and what is available for routine clinical use. In addition, the wide range of MRS sequences, parameters, and analysis choices can make the technique particularly difficult for nonexpert users. Nonetheless, MRS provides fundamental insights into cardiac metabolism in various cardiac disease states as well as in response to therapeutic intervention. It has the ability to dramatically advance our understanding of the pathophysiology and metabolic nature of a number of cardiac conditions, especially in patients with valvular heart disease,^9^^,^^28^ heart failure, ischemic heart disease, and other cardiomyopathies.^34^

#### ^1^H-MRS for assessing lipid metabolism

Myocardial triglycerides (MTGs) can be assessed with the use of cardiac ^1^H-MRS, which uses the abundant hydrogen (^1^H) protons. For ^1^H-MRS, data are typically acquired at breath hold during diastole from a single voxel (14-16 mL) localized in the myocardial septum (Figure 4A) and take 10 to 15 minutes to acquire.^35^ ^1^H-MRS is increasingly used in research studies but has not yet fulfilled its promise in clinical cardiology, because of a variety of practical challenges, including longer scan times to obtain sufficient signal-to-noise ratio (SNR) for detection of low-concentration metabolites and other technical considerations with data acquisition, postprocessing, and analysis. Many of these challenges are being overcome with higher magnetic field strengths and new MRS acquisition techniques; for example, ^1^H-MRS has been performed at our center in only 6 to 7 breath holds at 3.0-T with the use of a stimulated echo sequence, allowing reliable and quick quantification of myocardial lipids.^35^ Other metabolites (eg, creatine and choline) are clinically relevant but more challenging to quantify because of their relatively low concentrations (∼10 mmol/L) and because of cardiac motion. To quantify these, more sophisticated acquisition methods and in-house expertise are required. Our group has shown the feasibility of ^1^H-MRS in detecting low concentration metabolites by adding a water suppression cycling technique to single-voxel spectroscopy sequences at 3.0-T in patients with AS.^36^

Various clinical studies have assessed the presence of myocardial steatosis with the use of ^1^H-MRS and examined its functional associations in obesity, type 2 diabetes mellitus,^37^ and normal individuals when subjected to prolonged exercise and diet restrictions.^38^ In severe AS, our group has demonstrated pronounced steatosis (2-fold higher compared with control subjects) in both symptomatic and asymptomatic patients associated with impaired LV strain (Figure 4B).^28^

Further research is required to correlate the degree of steatosis with the stage of valve disease, its overall prognostic value and whether modulating steatosis could be a potential therapeutic option in AS. Although there have been single-center studies using ^1^H-MRS in various cardiac diseases, there is a need for multicenter studies to validate those findings and allow establishment of uniform standards for coil production, image acquisition protocol, and data analysis. With the current pace of research, this is achievable as already proven by the adoption of ^1^H-MRS in noncardiac imaging such as clinical brain and cancer imaging.^39^ Similarly, the use of ^1^H-MRS in clinical cardiology is potentially visageable in the near future.

### Pyruvate metabolism in the heart

**Figure 5:**
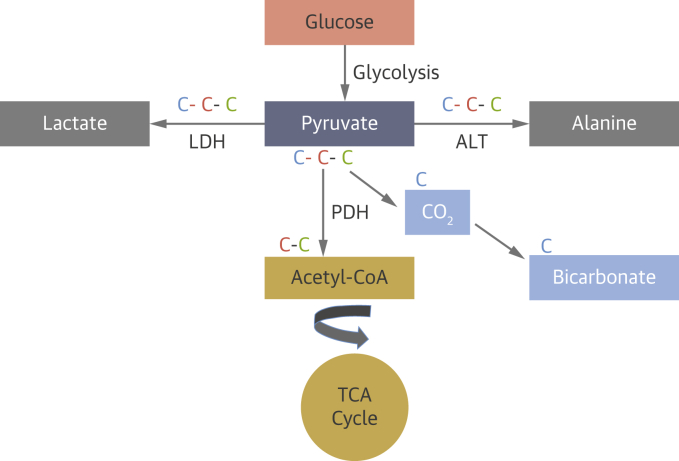
Pyruvate Metabolism Hyperpolarization imaging helps in investigating the metabolic fate of the first carbon atom **(blue)** when pyruvate is metabolized to alanine via alanine transaminase (ALT), to lactate via lactate dehydrogenase (LDH), and to bicarbonate via pyruvate dehydrogenase (PDH). The second and third carbon atoms **(red, green)** enter the tricarboxylic acid cycle (TCA) when incorporated into acetyl coenzyme A (acetyl-CoA).

Pyruvate is rapidly taken up by cardiomyocytes and metabolized through 3 major pathways. It can be converted through anaerobic metabolism to lactate via lactate dehydrogenase or to alanine via alanine transaminase, or it can be metabolized via pyruvate dehydrogenase (PDH) to acetyl-CoA and CO_2_, which is in dynamic equilibrium with bicarbonate via the enzyme carbonic anhydrase (Figure 5).

**Figure 6:**
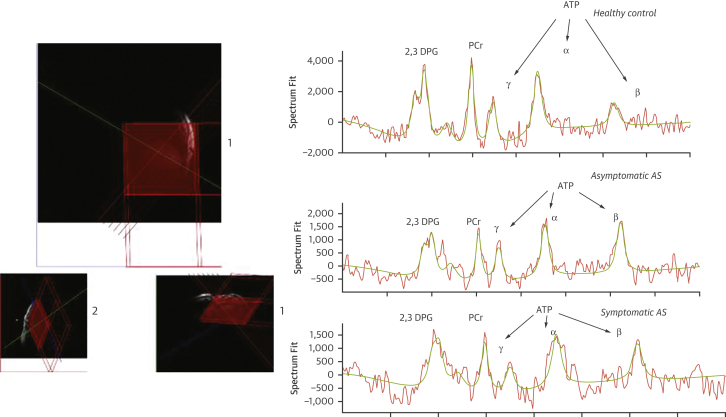
^31^P Spectra of the Heart From a Patient With Aortic Stenosis Image on the **top left** shows the position of the voxel in the interventricular septum (short-axis view). Overlaid with spectra showing 2,3-diphosphoglycerol (DPG), phosphocreatine (PCr), and adenosine triphosphate (ATP) peaks derived from postprocessing of phosphorous (^31^P) magnetic resonance spectroscopy data in healthy volunteers and asymptomatic and symptomatic aortic stenosis (AS).

#### Hyperpolarized ^13^C imaging for assessing pyruvate metabolism

The very low signal from most molecules involved in pyruvate metabolism significantly hampers their assessment with MRS. This may be overcome, however, with hyperpolarized ^13^C imaging,^40^ where the MR-active nuclei such as ^13^C are mixed with a low concentration of free electrons and the sample is irradiated with microwaves in a high magnetic field (>3.0-T) and at low temperature (∼1 °K). The hyperpolarizer system allows sample dissolution to temporarily maintain the high signal in solutions with a physiologic temperature and pH suitable for injection. After injection, the enhanced signal, though short-lived, about 1-2 minutes, can then be use to study flux through metabolic pathways in vivo.^41^

The metabolism of the injected [1-^13^C]pyruvate provides information on key metabolic reactions (ie, lactate dehydrogenase, PDH, and alanine transaminase).^42^ Other molecules have also been successfully studied, including [2-^13^C]pyruvate^43^ for investigation of metabolism through the TCA cycle, [1,4-^13^C_2_]fumarate^44^ for assessment of cellular necrosis, and ^13^C-bicarbonat,^45^ for in vivo assessment of extracellular pH.

Hyperpolarized ^13^C spectroscopy has enabled the assessment of pyruvate metabolism in vivo in humans.^46^ The technique potentially provides a window on several important metabolic processes that are essential to cardiac function and vary during differing disease processes, including diabetes,^47^ dilated cardiomyopathy,^40^ ischemic heart disease,^48^ cardiac hypertrophy, and HF.^49^

In the pressure-overloaded myocardium, there is proposed to be an increase in glycolysis despite a normal level of flux through PDH, leading to increased incorporation of glycolytically derived pyruvate into lactate.^16^ Histologic studies in hypertrophied rat hearts have demonstrated that this mismatch between glycolysis and glucose oxidation is the consequence of increased pyruvate carboxylation and lower flux through PDH, resulting in the increased lactate production.^50^ Use of hyperpolarized ^13^C imaging in the pressure-overloaded state in vivo could remarkably improve our understanding of the metabolic alterations and their effect on cardiac function.

The clinical use of hyperpolarized ^13^C imaging is in its infancy, with only selected centers having the capability to run cardiac scans in humans. The potential for the use of hyperpolarized imaging has largely been demonstrated in the preclinical setting,^16^ but the feasibility of using hyperpolarized ^13^C technique in the setting of human cardiovascular disease has been achieved.^51^

An alternative metabolic imaging technique to study TCA cycle metabolites is deuterium MRS (DMRS). This has recently been used in combination with an infusion of deuterium-labeled glucose or acetate by Wang et al^52^ in rat hearts to determine the rates in vivo of glucose metabolism and the TCA cycle, which dominates mitochondrial ATP production in supporting cardiac function. Though not yet tested in humans, DMRS could be valuable for investigating the metabolic shift from preferred FAO to glucose oxidation under stress and diseased conditions.^53^ To develop the DMRS technique for clinical translation, further research is needed to understand the relationship between imaging measures and cardiac pathophysiology in human patients.

The number of clinical applications for these techniques is growing rapidly, especially in assessing ischemia, perfusion, and viability. Clinical studies are ongoing to establish the clinical efficacy.

### High-energy phosphate metabolism in AS

Pressure-overload LVH increases the energetic cost of mechanical work, and when this is at the severe end of the spectrum the resulting mismatch in myocardial energy supply and demand may contribute to the development of HF. The PCr/ATP ratio, an index of cardiac bioenergetic state, is reduced in nonhuman animal models of myocardial hypertrophy^10^ and in human LVH^9^ and HF.^54^ It not only correlates with the degree of cardiac hypertrophy and accompanying LV dysfunction,^55^ but also has been shown to be a superior predictor of mortality.^4^^,^^56^

Creatine plays an important role in the buffering and transport of chemical energy to ensure that supply meets the dynamic demands of the heart. Gradual loss of myocardial total creatine content and a corresponding reduction in CK activity is observed in HF,^57^^,^^58^ animal models of cardiac hypertrophy,^59^ and hypertrophied human myocardium from patients with AS.^60^

Reduced CK flux has been shown to limit contractile reserve and contribute to the transition to systolic failure in hypertrophied hearts.^11^^,^^61^ As the energetic changes appear to occur early in the disease process, being present in moderate AS, it seems likely that energetic impairment precedes LV systolic dysfunction in the pressure-overload state.^61^^,^^62^

#### ^31^P-MRS for assessing high-energy phosphate metabolism

^31^P-MRS allows the in vivo quantification of phosphorus (^31^P)–containing metabolites involved in energy metabolism, such as PCr and ATP (Figure 6). With the use of ^31^P-MRS, various indices of the CK system have been measured to assess mitochondrial energetics, including the PCr/ATP ratio and forward CK flux.^63^ The average PCr/ATP ratio in a healthy human heart is 2.03 ± 0.38 in the literature,^61^^,^^64^ but the absolute value is dependent on the sequence used.^63^^,^^65^^,^^66^ Therefore, institution-specific reference ranges are generally used at present. Cardiac ^31^P-MRS in humans is typically performed at higher magnetic field strengths of 1.5-T to 3.0-T and takes 10 to 30 minutes to acquire. Although the variability of MRS is often cited as a limitation, modern techniques typically yield a variability of 13% for PCr/ATP ratios, which is in the same range as LV volumes and functional assessment on CMR imaging and echocardiography.^67^, ^68^, ^69^ Future developments and technical advances of MRS at higher field strengths aim to deliver substantial further improvements to make the method valuable for clinical practice.

Studies have shown that PCr/ATP is a better predictor of long-term survival than New York Heart Association functional class or LV ejection fraction in several cardiac conditions, including dilated cardiomyopathy,^70^ hypertrophic cardiomyopathy,^71^ HF with preserved ejection fraction,^72^ and ischemic heart disease.^73^ CK reaction rate and flux also can be probed with the use of ^31^P-MRS, which provides a more accurate assessment of cardiac energetic state than PCr/ATP alone.^61^

We^74^ and others^75^ have demonstrated that both PCr/ATP ratio and CK flux are reduced in symptomatic AS patients, being lowest in those with associated systolic failure,^61^ and correlates with LV end-diastolic pressures and end-diastolic wall stress, in line with previous histologic animal and human studies.^59^^,^^60^ Studies have also shown that impaired energetics in AS are reversible following relief of pressure overload and hypertrophy regression.^76^^,^^77^ The method and sample spectra comparing the PCr/ATP from a healthy volunteer, and asymptomatic AS patient, and a symptomatic are shown in Figure 5.

## Other Pressure-Overload LV Disorders

These metabolic changes secondary to reduced myocardial FA metabolism are not exclusive to AS; other pressure-overload LV disorders, such as hypertensive heart disease, show a similar maladaptive substrate switch and consequential energy-hungry state.^78^^,^^79^ As in AS, these phenomena precede the increase in LV mass and are potentially responsible for decreased myocardial efficiency and subsequent heart failure.^80^

The basis of these metabolic changes in the pressure-overloaded heart is substantial metabolic reconfiguration, including substrate utilization switch from FAs to glucose, uncoupling of glucose uptake from oxidation with enhanced glycolysis,^15^ FAO down-regulation,^16^ impaired mitochondrial respiration,^22^^,^^81^ and decreased mitochondrial/cytosolic CK flux,^61^ together with loss of metabolic flexibility to stress.^7^ These are partly driven by nuclear receptor and transcriptional coregulator signaling circuits orchestrating fuel selection and mitochondrial oxidative capacity, in which peroxisome proliferator–activated receptor (PPAR)-α,^82^ an FA ligand–binding master transcription factor promoting FAO, plays a critical role along with interacting regulators of oxidative metabolism such as PPARγ coactivator (PGC)-1α.^83^ PPARα not only plays a key role in the transcriptional control of substrate switching,^84^ but artificial ligands for PPARα, such as fenofibrate, also protect against endothelin-induced cardiac hypertrophy and failure,^85^ and cardiac function is seen to be severely damaged in PPARα-null mice during pressure overload.^86^

Overall, decreased FAO clearly contributes to the reappearance of the fetal metabolic pattern in hypertrophied and failing hearts that leads to increased reliance on glycolysis^87^ combined with up-regulation of anaplerosis to maintain TCA flux,^17^ and it slightly improves myocardial oxygen efficiency, but this metabolic profile is inefficient in utilizing carbon substrates for ATP production during increased energy demand, leading to impaired myocardial energetics and depletion of contractile reserve.^7^^,^^18^

Accompanying this switch is also an imbalance between FA uptake and FA oxidative metabolism, leading to intracellular cardiac lipid accumulation. This accumulation provides a source for nonoxidative metabolism to diacylglycerol and ceramide, potentially resulting in lipotoxicity, apoptosis, and cardiac dysfunction.^14^^,^^21^^,^^26^

CMR is a well-established technique to assess cardiac function, morphology, valve anatomy, and function in patients with AS.^88^ Late gadolinium enhancement and T1 mapping techniques are able to assess myocardial fibrosis that occurs as a result of structural remodeling in AS and could offer prognostically important information. MRS allows in vivo assessment of myocardial metabolism^30^ and unlike other imaging modalities such as positron emission tomography or single-photon emission computed tomography, it has the combined advantages of providing molecular information while also being free of ionizing radiation and not requiring the administration of contrast agents.^89^ Table 1 presents a concise comparison between PET and CMR.

**Table 1:** Brief Comparison of Main Pros and Cons of Cardiac Magnetic Resonance and Positron Emission Topography

	Cardiac Magnetic Resonance	Positron Emission Topography
Anatomic information	Extensive	Limited
Ionizing radiation	None	Up to 7-9 mSV
Spatial and temporal resolution	Superior	—
Technical challenges	Widely available	Availability restricted to few centers
Cardiac diseases	Well-established role in a spectrum of cardiac diseases, including DCM, IHD, and hypertrophy	Clinical use mainly limited to assessment of myocardial viability in IHD, or myocardial inflammation in cardiac sarcoid
Myocardial metabolism	MRS well established in assessing FA, lipid, and pyruvate metabolism as well as myocardial energetics	Mainly glucose metabolism using ^18^FDG; other tracers like ^11^C-palmitate still experimental

DCM = dilated cardiomyopathy; FA = fatty acid; FDG = fluorodeoxyglucose; IHD = ischemic heart disease; MRS = magnetic resonance spectroscopy.

## Limitations of MRS

MRS has so far primarily been used as a research tool because of relatively low spatial resolution and wide variability in acquisition and postprocessing techniques, with expertise currently restricted to large centers. Several technical advances have been made in recent years to overcome these challenges, including the use of advanced coils, sophisticated acquisition sequences, quicker MRS postprocessing tools, and higher magnetic field strengths for faster imaging and data analysis. A close collaboration between basic scientists and clinicians, as well as a strong partnership between academia, industry, and funding agencies is required to progress further in this area. Table 2 presents a brief overview of the limitations and ability for clinical translation for various available MRS techniques.

**Table 2:** Overview of Different Available Magnetic Resonance Spectroscopy Techniques, Their Limitations, and Feasibility to Clinical Translation

MRS Nuclei/Technique	Main Use	Strengths and Limitations	Potential Solution	Clinical Translation
^1^H-MRS	Can detect and quantify cardiac lipids, creatine, and choline	High sensitivity and abundance	Use of advanced surface coils and higher field strengths	Already used in clinical brain and cancer imaging
		Low spatial resolution		Could be adopted for use in clinical cardiac imaging in specialized centers
^31^P-MRS	Can detect metabolites involved in high-energy phosphate metabolism in the heart	High sensitivity and 100% natural abundance	Use of advanced surface coils and higher field strengths	Could be adopted for use in clinical cardiac imaging in specialized centers
	Indirectly deduce intracellular pH	High quality spectra obtainable even at low field strengths		
^13^C-MRS	Can detect and quantify pyruvate metabolism metabolites	Low sensitivity and abundance (1%)	Use of hyperpolarized MRS technique increases signal	Already used in prostate cancer imaging
			Higher field strengths	
^2^H-MRS	Can detect TCA cycle metabolites	Low sensitivity and abundance (1%)	Still in developmental stage; not yet used in humans	Not ready for clinical use
		Currently only performed at ultra-high field strength and in experimental models		
^23^Na-MRS	Can detect tissue viability	Very low sensitivity and concentration	Advanced sequences and coils to improve detection	Not ready for clinical use
		Long acquisition times	Higher field strengths	

^13^C = carbon; ^1^H = proton; ^2^H = deuterium; MRS = magnetic resonance spectroscopy; ^23^Na = sodium; ^31^P = phosphorous; TCA = tricarboxylic acid.

For MRS to be adopted as a clinical imaging tool to advance patient care, its clinical utility will have to be tested in subsequent larger studies. Critical to this is the demonstration of reproducibility, which will necessitate the establishment of uniform standards for coil production, image acquisition protocol, and data analysis that enable multicenter studies. To facilitate this, early small multicenter studies are required to validate the findings from single-center trials, and indications supported by those small multicenter trials can then be carried into larger studies. These larger studies will not only evaluate safety and efficacy end points, but also collect information on clinical impact and cost-effectiveness. The true potential of MRS techniques lies in their rapid progress to clinical translation for patient care, and with the ongoing advances and improvements in MRS techniques this is highly plausible in the near future.

## Future Directions

Because lipid metabolism seems to underpin both the metabolic and the hypertrophic mechanisms seen in the pressure-overload state, this may be a common pathway to target as a therapy. MRS, with its recent improvements in technique and advanced processing systems, will prove to be of clinical value in establishing the link between abnormal metabolism and progression from compensated hypertrophy to HF. It could potentially identify metabolic biomarkers to monitor progression of the disease and help in risk stratification in AS. In this way, it not only will be helpful in guiding decision making for valve replacement in addition to currently available imaging tools, but also could help in exploring avenues for precision metabolic therapy.

Thus, a therapeutic approach that alters myocardial substrate selection may target both the cardiac metabolic and the structural effects of pressure overload and is likely to be effective in treating cardiac dysfunction in AS and other pressure-overload disorders. PPAR agonists are one such group of drugs, especially PPARα, which play a central role in the FAO signaling system as well as control lipid homeostasis.^90^ The ability of PPARα receptors to respond to distinct metabolic cues provides a potential mechanism to maintain a balance between FA breakdown and storage, and their down-regulation in the pressure-overload hypertrophy state has been shown to have deleterious effects.^91^ Fibrates (PPARα agonists) are one such group of drugs that hold promise with their ability to up-regulate FAO in cardiac myocytes and reduce lipotoxicity in pressure-overload hypertrophy. Another group that may hold potential are PPARγ agonists, Thiazolidinediones, that help in adipogenesis and redirect excess free FAs to adipose cells preventing lipotoxicity. In the heart, they also oppose inflammatory pathways and act as a growth suppressor.

The successful translation of therapies targeting cardiac substrate alterations in AS will require a deeper understanding of the interplay between cardiac substrate metabolism, lipid deposition, energy generation, and cardiac function, especially in those with asymptomatic moderate to severe AS and preserved ejection fraction.

## Conclusions

Changes in myocardial metabolism in pressure-overload LVH can be identified by the unique noninvasive techniques of MRS. These are important for understanding the pathophysiologic processes, identifying those most at risk of decompensation and for developing new therapeutic targets.

In hypertrophied AS hearts, myocardial metabolic changes occur early, preceding LV decompensation. This provides an opportunity for earlier identification of metabolic maladaptation in AS (before LV functional decompensation) and the potential to intervene to delay or prevent decompensation. This could benefit large numbers of patients (particularly the elderly), and provide an alternative to the current end-stage mechanical solution of aortic valve replacement.

## Funding Support and Author Disclosures

Dr Monga, Prof Myerson, and Prof Mahmod have received research grant support from the British Heart Foundation for research into metabolic treatments in aortic stenosis (clinical research training fellowship number: FS/18/17/33514). Prof Valkovič is supported by a Sir Henry Dale Fellowship from the Wellcome Trust (#221805/Z/20/Z). Prof Tyler is supported by a British Heart Foundation Senior Research Fellowship (FS/19/18/34252). Prof Lygate has received funding from British Heart Foundation Programme grant RG/18/12/34040. Prof Rider is supported by a British Heart Foundation fellowship (FS/16/70/32157). Prof Myerson’s research is supported by the National Institute for Health Research (NIHR) Oxford Biomedical Research Centre. Profs Tyler, Rider, and Neubauer have received support from the Oxford NIHR Biomedical Research Centre and the Oxford British Heart Foundation Centre of Research Excellence.
